# Hydrogen Production via Methane Decomposition over Alumina Doped with Titanium Oxide‐Supported Iron Catalyst for Various Calcination Temperatures

**DOI:** 10.1002/open.202300173

**Published:** 2023-12-12

**Authors:** Hamid Ahmed, Mohammed F. Alotibi, Anis H. Fakeeha, Ahmed A. Ibrahim, Ahmed E. Abasaeed, Ahmed I. Osman, Abdulrahman S. Al‐Awadi, Naif Alarifi, Ahmed S. Al‐Fatesh

**Affiliations:** ^1^ College of Engineering King Saud University P.O. Box 800 Riyadh 11421 (Kingdom of Saudi Arabia; ^2^ Institute of Refining and Petrochemicals Technologies King Abdulaziz City for Science and Technology (KACST) P.O. Box 6086 Riyadh 11442 Kingdom of Saudi Arabia; ^3^ School of Chemistry and Chemical Engineering Queen's University Belfast Belfast BT9 5AG Northern Ireland (UK

**Keywords:** Hydrogen Production, Methane Decomposition, Iron Catalyst, TiO_2_- Al_2_O_3_, Carbon nanotubes

## Abstract

The decomposition of methane has been chosen as an alternative method for producing hydrogen. In this study, 20 % Fe was used as the active metal part of the catalyst. To better comprehend the impact of the supporting catalytic properties, alumina and titania‐alumina composite were investigated as supports. Iron‐based catalysts were prepared by impregnation method and then calcined at different temperatures (300 °C, 500 °C, and 800 °C). The catalysts were examined at 800 °C under atmospheric pressure with a 15 mL/min total flow rate and 2 : 1 CH_4_ to N_2_ feed ratio. The textural and morphological characteristics of the fresh calcined and spent catalysts were investigated. The catalytic activity and stability data demonstrated that Fe supported over TiO_2_‐Al_2_O_3_ calcined at 500 °C performed the best of all evaluated catalysts with a more than 80 % hydrogen yield. The Raman spectra result showed that graphitic carbon was produced for all used titanium dioxide catalysts. Moreover, according to transmission electron microscopy (TEM) results, the carbon deposited on the catalysts’ surface is carbon nanotubes (CNT).

## Graphical abstract

Iron metal was used for hydrogen production via methane decomposition using alumina as support; furthermore, to overcome the drawback of alumina, titanium dioxide was added at 20 %, considering various calcination temperatures. The results show that adding 20 % of TiO_2_ to alumina enhances the activity of catalysts for hydrogen production and stability. Carbon was produced as carbon nanotubes (CNT).

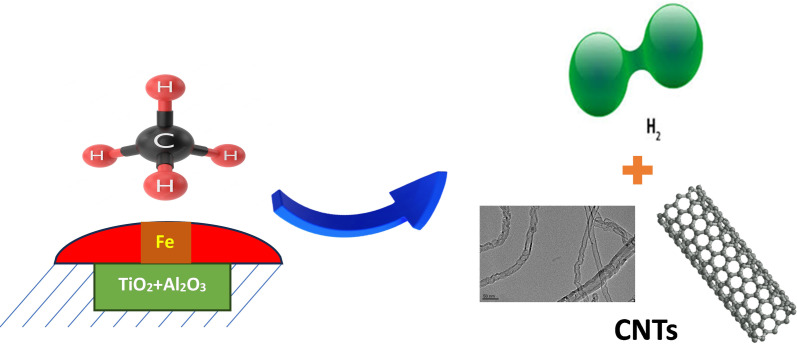

## Introduction

Hydrogen is one of the few energy carriers that can provide a high standard of living without harming the environment.^[1]^ Much attention has been paid to its potential for fuel cell and internal combustion engine applications. It is also recognized as the twenty‐first century‘s primary energy transporter and fuel. It has significant potential in various industrial applications that can lessen detrimental environmental impacts.[^2^, ^3^] Moreover, hydrogen is widely used in chemical processes; in particular, it is utilized as a raw material to manufacture olefins and alcohol through hydrogenation processes.[^4^, ^5^] Although methane is a greenhouse gas leading to global warming, it can be an essential source for hydrogen production.[^6^, ^7^, ^8^] A methane molecule has four very strong C−H bonds created through sp3 hybridization. This gives it a high level of stability and inactivity, with an energy of around 435 kJ mol^−1^. Because of this, breaking down methane requires a high temperature of over 1200 °C and is an endothermic process. Therefore, a catalyst is always used to reduce the activation energy and lower the reaction temperature to below 400 °C, resulting in a significant product yield.[^9^, ^10^, ^11^] Hydrogen and added‐value carbon are formed from methane gas. Steam reforming of methane (SRM), coal and biomass gasification, dry reforming, and partial oxidation of methane (POM) are all methods for producing hydrogen.^[12]^ However, In the processes of (SRM) and (POM) reactions, carbon dioxide (CO_2_) and carbon monoxide (CO) are simultaneously produced.^[13]^ As a result, green and sustainable production of high‐purity H_2_ and reduced greenhouse gas emissions are advantageous and crucial.[^14^, ^15^, ^16^] The catalytic decomposition of methane (CDM) is offered as a green, one‐step method that makes hydrogen production practical and forms various types of carbon amorphous, graphitic, and carbon nanotubes (CNTs).[^18^, ^19^, ^20^] The growth mechanism of CNTs can be categorized into tip growth and base growth. The dominant type depends on the interaction between the catalyst metal and support weakness. If the interaction is weak, then tip growth occurs, where the growing CNT lifts the catalyst particle. Strong supports lead to base growth, where the growth mechanism proceeds through an open tip.^[17]^

(1) $${CH}_{4}\to C+2\;H_{2}\;\Delta H=74.9\;KJ/mol$$

The structure, texture, and morphologies of the carbons formed in the CDM reaction depend on the reaction conditions, such as pressure, temperature, catalysts, and gas phase composition. These carbons have numerous commercial applications, such as electrodes, catalytic supports, and direct catalysts.^[26]^ Nickel and Cobalt‐based catalysts′ activity in the hydrogen production from methane decomposition is higher than that of Fe; nevertheless, faster catalyst deactivation occurs through carbon encapsulation and sintering at temperatures exceeding 650 °C.^[27]^ To make the process of converting methane into hydrogen more cost‐effective and environmentally friendly, an affordable catalyst that doesn′t need regeneration of spent catalyst and carbon materials mixtures is necessary. Iron is a potential option for this because its partially filled 3d orbitals facilitate hydrocarbon dissociation by partially accepting electrons. Iron is also more cost‐effective than nickel and has a higher melting point, enabling Fe catalysts to operate at higher temperatures than Ni catalysts. As a result, thermodynamic conversion in CDM, an endothermic reaction is improved.[^19^, ^28^, ^29^] Various literature sources report on the decomposition of methane using Fe‐based materials, and this information has been gathered in Table 1.

**Table 1:** Literature review of CDM over Fe‐based catalysts.

Sample	Preparation Method	Loading (%)	Reaction Temperature (°C)	Methane Conversion (%)	Hydrogen Yield (%)	Ref.
Fe/CeO_2_‐ZrO_2_	impregnation	15	700	–	83	[21]
Ni‐Fe‐Zn/IM‐5	Co‐precipitation	5	670	65	–	[22]
Iron Ore	–	75.3	800	35	47	[23]
Fe_2_O_3_/Y_2_O_3_‐Al_2_O_3_	Sol‐Gel	3	750	29	–	[24]
Fe/La_2_O_3_+ZrO_2_	impregnation	40	800	79	84	[25]

To enhance the activity and stability of the Fe‐based catalyst, various promoters, supports, synthesis processes, reactor types, and operating conditions have been investigated to promote a longer catalyst lifetime; these investigations aim at enhancing the surface area of the catalyst, the active metal dispersion on the support, and the metal‘s electronic states while decreasing catalyst sintering.[^30^, ^31^, ^32^] Single oxide materials, such as CeO_2_,^[8]^ SiO_2_,^[33]^ Al_2_O_3_,^[34]^ MgO,^[35]^ and ZrO_2_,^[25]^ are widely utilized as supports for Fe‐based catalysts in the CDM process. For example, Al_2_O_3_ has been extensively investigated as support for Fe‐based catalysts because of forming a strong metal–support interaction, which can enhance the reactivity by facilitating the CH_4_ dissociation over the metallic Fe active site.^[34]^ Using composite oxides as support materials is common, alongside single oxides, because they can overcome single oxide limitations and enhance the performance of catalysts. One drawback of using alumina as a single oxide supporting material is its low mechanical strength.^[2]^ Titania, or titanium dioxide, occurs naturally in three crystal phases: rutile, anatase, and brookite. Rutile is the most stable of the three, whereas anatase and brookite will undergo irreversible exothermic reactions when exposed to heat and eventually convert to rutile. Rutile and anatase‐phase TiO_2_ are commonly used as a support for catalysts. Rutile‐/anatase‐phase TiO_2_ has been successfully utilized as a deposition site for non‐noble metal species such as Cr, V, and Fe.[^36^, ^37^] However, pure titanium has a low surface area (70 m^2^/g) and pore volume (0.3 mL/g); titanium has a wide range of uses in nanoscience as a photo‐catalyst; titanium incorporation into alumina to synthesize alumina‐titanium composites is increasingly prevalent, with significant improvements in alumina‘s thermal stability, activity, and mechanical strength, as well as a greater surface area of the alumina‐titanium mixture compared to titanium alone. The Al_2_O_3_‐TiO_2_ system has many applications, including ceramic powder, membrane, catalyst, and catalytic support.[^38^, ^39^]

The calcination temperature affects the catalyst‘s structural characteristics, influencing CH_4_ conversion activity. Increasing calcination temperature increases particle size, pore diameter, and crystal size while decreasing pore volume and specific surface area. The CH_4_ conversion is favored by increasing the calcination temperature up to a certain point, but after that point, it causes a reduction in its catalytic activity, mainly caused by the agglomeration of the particles and reduction in the specific surface area.^[40]^ At higher calcination temperatures, particle agglomeration occurs, resulting in larger crystal sizes and a reduction in specific surface area due to the collapse of the porous structure.^[41]^ For example, Al‐Fatesh and his colleagues^[41]^ examined iron catalysts supported on alumina prepared with three different methods and used two calcination temperatures. They found that regardless of the preparation method, the catalytic activity was high when calcined at 500 °C for the sol‐gel method. In addition, using the impregnation method catalyst increased activity when calcined at high temperatures. According to the activity results, the catalyst is stable for up to six hours on stream. They also observed that the amount of carbon deposition depends on the preparation method and calcination temperature.

**Figure 1:**
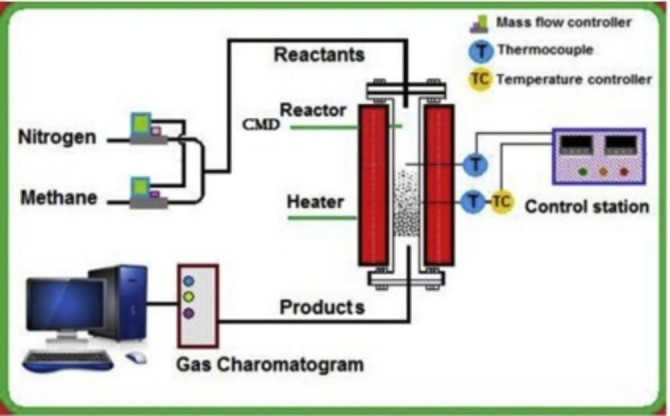
Schematic diagram of the experimental setup for catalytic hydrogen production.

This work prepared an iron‐based catalyst to produce hydrogen in the CDM process using the wet impregnation method. Single catalyst support of alumina and binary catalyst support of alumina and titanium oxide were used as the support material. The reason for using titanium oxide is to overcome the drawback of the low mechanical strength of the alumina. The catalysts were calcined at various temperatures (300 °C, 500 °C, and 800 °C). The activity and stability of methane decomposition operated at 800 °C under atmospheric pressure in the presence of inert N_2_ gas were investigated. Various characterization techniques were employed to substantiate the findings. Figure 1 illustrates the schematic diagram of the experimental setup used in this study.

## Results and Discussion

### Structure Analysis

The N_2_‐physisorption analysis was performed for Al_2_O_3_ and TiO_2_‐Al_2_O_3_‐supported Fe catalysts at various calcination temperatures (300 °C, 500 °C, 800 °C); the results are reported in Table 2.

**Table 2:** Textural properties of fresh and used catalysts calcined at different temperatures.

Catalyst Name	Fresh Catalyst			Used Catalyst		
	BET(m^2^/g) Surface area	Pore Volume (cm^3^/g)	Pore Size (nm)	BET(m^2^/g) Surface area	Pore Volume (cm^3^/g)	Pore Size (nm)
Fe/Al‐300	249.89	0.63	9.20	114.39	0.32	10.89
Fe/Al‐500	224.37	0.71	11.20	111.22	0.37	12.50
Fe/Al‐800	149.00	0.65	16.20	80.54	0.32	15.50
Fe/Ti‐Al‐300	283.52	0.76	10.60	110.59	0.55	20.25
Fe/Ti‐Al‐500	256.74	0.90	13.20	77.00	0.44	23.49
Fe/Ti‐Al‐800	128.68	0.81	27.40	86.79	0.52	24.65

**Figure 2:**
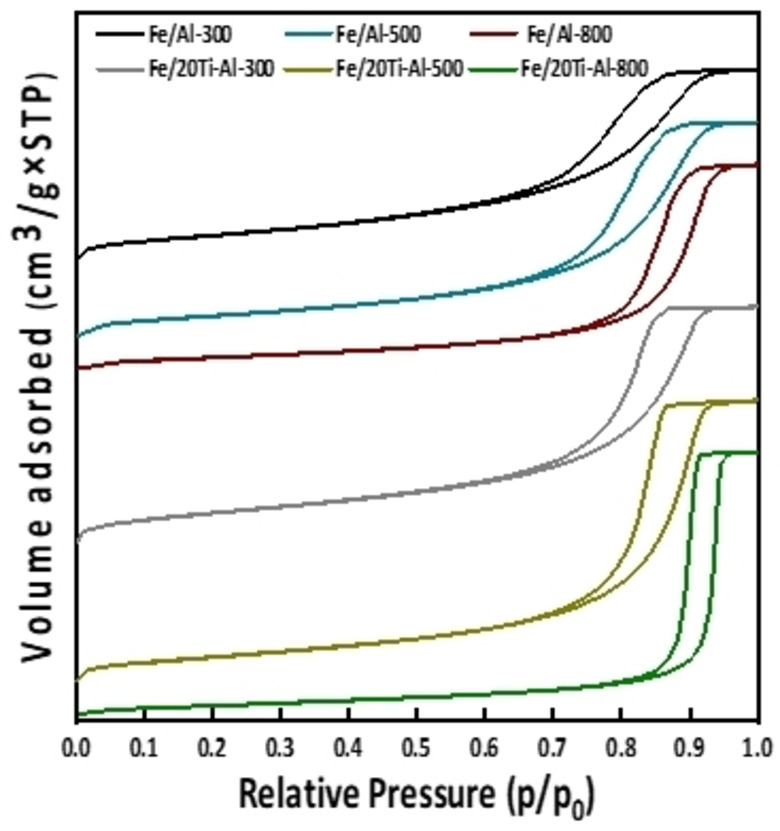
N_2_ adsorption‐desorption isotherms for fresh iron‐based catalysts loaded on individual and binary support calcined at various temperatures.

Incorporating titanium oxide into alumina improved the support‘s pore volume and surface area, except for the catalyst calcined at 800 °C. For instance, the surface areas increased from 249.89 and 224.37 m^2^/g for unmodified Fe/Al_2_O_3_ to 283.52 and 256.74 for Fe/(TiO_2_‐Al_2_O_3_) calcined at 300 °C and 500 °C, respectively. Also, the pore volumes of Fe/ (TiO_2_‐Al_2_O_3_) calcined at 300 °C and 500 °C improved to 0.76 cm^3^/g and 0.90 cm^3^/g, respectively, in contrast to unmodified samples calcined at the same temperatures where the obtained values are 0.63 cm^3^/g at 300 °C and 0.71 cm^3^/g at 500 °C. Incorporating TiO_2_ into the Al_2_O_3_ boosts the pore volume and specific surface area. Figure 2 exhibits the N_2_ adsorption‐desorption isotherms and pore size distribution of Fe‐containing catalysts. All of the adsorption‐desorption isotherms for the catalysts are of type IV, with H3 hysteresis loops, a characteristic of mesoporous materials according to the IUPAC classification. Nevertheless, the mesoporosity was significantly enhanced by incorporating TiO_2_ into the Al_2_O_3_ support, as shown by the expansion of the hysteresis loops in Figure 2. This indicated that the interaction between TiO_2_ and Al_2_O_3_ could produce additional pores through the partial collapse of the Al_2_O_3_ matrix during calcination.

### Temperature‐Programmed Reduction (TPR)

**Figure 3:**
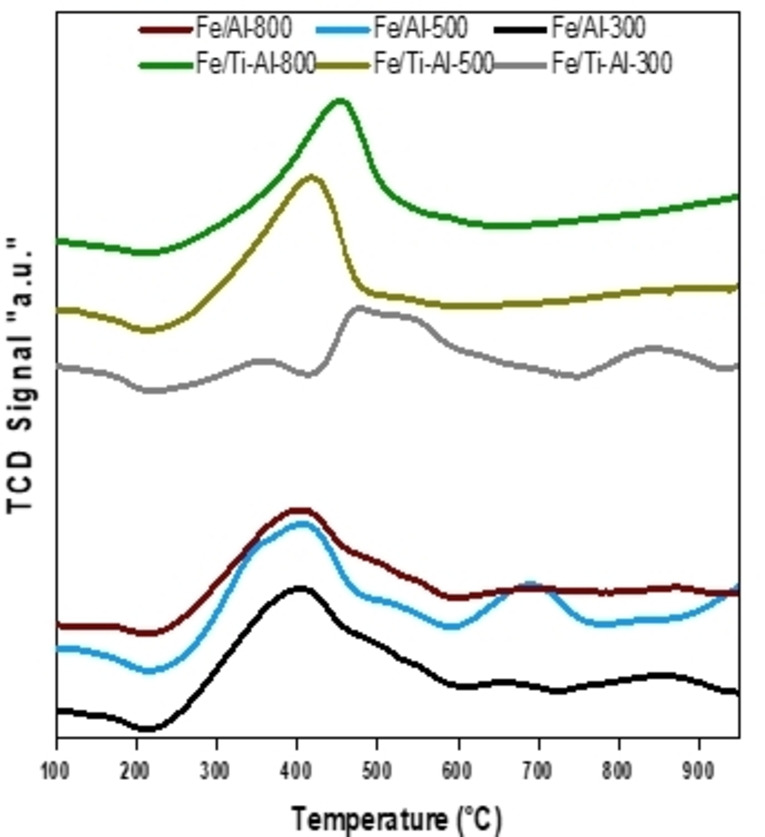
H_2_‐Temperature‐Programmed Reduction spectra for fresh individual and binary‐supported iron catalysts calcined at different temperatures.

H_2_ temperature‐programmed reduction (H_2_‐TPR) has been considered an effective technique for identifying various species in solid solutions. Generally, the peak temperature of the TPR profile indicates the interaction of the combined support and active metal.^[42]^ The results of a temperature scan with hydrogen as the reducing agent are shown in Figure 3.

Relative TPR patterns of supported catalysts are somewhat diverse. The Fe/Al_2_O_3_ catalysts showed two peaks appearing at approximately 350–470 °C (maximum at 400 °C) and 650–750 °C (maximum at 700 °C); the first peak is attributed to the reduction of Fe_2_O_3_ to Fe_3_O_4_. In contrast, the second peak represents the transformation of Fe_3_O_4_ to Fe.^[33]^ Figure 3 also depicts the impact of TiO_2_ on the reducibility and the degree of metal support interaction of the freshly calcined Al_2_O_3_‐supported Fe catalysts. The H_2_‐TPR profiles of Fe/(TiO_2_‐ Al_2_O_3_) catalysts calcined at (300, 500, and 800 °C) showed only one distinct reduction peak ranging approximately from 370 to 500 °C, which was assigned to the reduction of Fe_2_O_3_→Fe_3_O_4_. Additionally, Fe/(TiO_2_+ Al_2_O_3_) reduction peaks are shifted towards higher temperatures than other Fe/Al_2_O_3_ catalysts. This observation could be attributed to higher interaction between Fe and (TiO_2_‐Al_2_O_3_) support, and also, the Fe_2_O_3_ particles were more uniformly distributed across the surface of the (TiO_2_‐Al_2_O_3_) support.^[25]^

Table 3 presents the degree of reduction (DR) for each catalyst to analyze the observation further. Notably, all catalysts have (DR) values below 100 %, indicating the absence of hydrogen spillover that usually occurs during the reduction of support material.

**Table 3:** The degree of reduction of the catalysts calcined at various temperatures.^[a]^

Catalyst Name	Total H_2_ consumption from the TPR (cm^3^/g* STP)	Degree of Reduction (%)
Fe/Al‐300	25.06	29.77
Fe/Al‐500	21.09	25.31
Fe/Al‐800	18.05	19.40
Fe/Ti‐Al‐300	14.84	16.49
Fe/Ti‐Al‐500	5.74	6.78
Fe/Ti‐ Al‐800	17.99	23.53

[a] Degree of Reduction (%)=(Hydrogen consumption during H_2_‐TPR/theoretical H_2_ required to complete the reduction).

### X‐Ray Diffraction (XRD)

**Figure 4:**
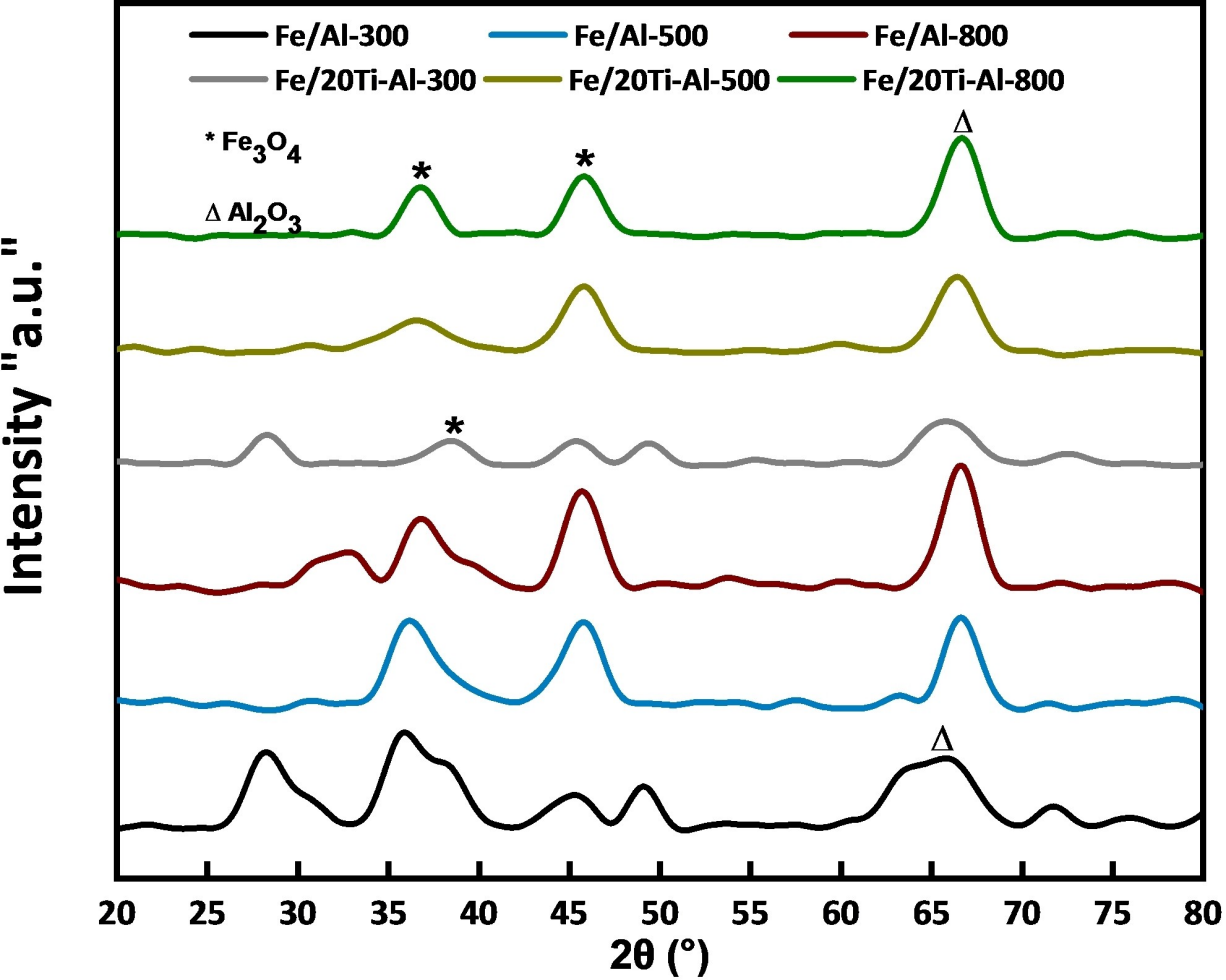
XRD patterns of the iron‐based catalysts loaded on individual and binary support calcined at different temperatures.

To explore the crystalline phases present in the prepared samples, powder X‐ray diffraction (XRD) was used. Figure 4 illustrates the diffraction angles of Fe/Al_2_O_3_ and Fe/(TiO_2_‐Al_2_O_3_) samples calcined at temperatures 300, 500, and 800 °C.

No discernible variation in the crystalline phases was found due to the calcination temperature. This suggests that the calcination temperature has minimal influence on the crystalline phase composition of the samples. The Fe/(TiO_2_‐Al_2_O_3_) catalyst calcined at 300, 500, and 800 °C has diffraction peaks at 2θ=36.8°, 45.8°, and 66.7°, while the Fe/Al_2_O_3_ catalyst has diffraction peaks at 2θ=35.9°, 45.8°, and 66.5°. The diffraction peaks at 2θ=66.5° and 66.7° correspond to Al_2_O_3_ (JCPDS: 00‐ 004–0875).^[32]^ The diffraction peaks at 2θ= 35.9°, 36.8°, and 45.8° are found in both catalysts related to Fe_3_O_4_. However, at a lower calcination temperature (300 °C), the peak related to hematite at 2*θ*=27° is also observed for both Fe/Al_2_O_3_ and Fe/(TiO_2_‐Al_2_O_3_) catalysts. In addition, the Fe/(TiO_2_‐Al_2_O_3_) catalysts didn't show any diffraction peaks corresponding to the TiO_2_ phase; the Rietveld method used in Figure S1 confirmed this result. This could be because of the low concentration of titanium oxide (20TiO_2_) when compared with alumina (80Al_2_O_3_) or another reason that the Fe_2_O_3_ particles were highly dispersed and strongly interacted with the (TiO_2_‐Al_2_O_3_) support.^[43]^

### Catalyst Activity Test

**Figure 5:**
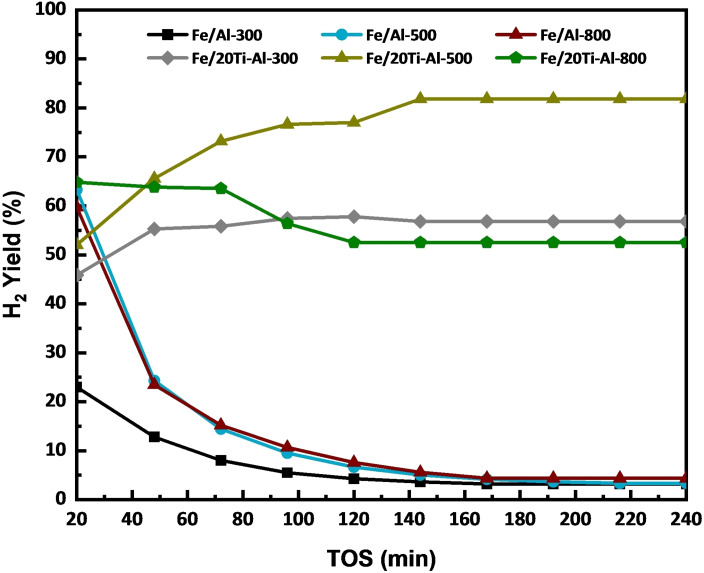
Time on Stream (TOS) of H_2_ Yield versus time on stream for iron‐based catalysts with individual and binary support at 800 °C, CH_4_/N_2_=2 : 1, P=1 atm, GHSV= 6 L g cat^−1^ h^−1^.

**Figure 6:**
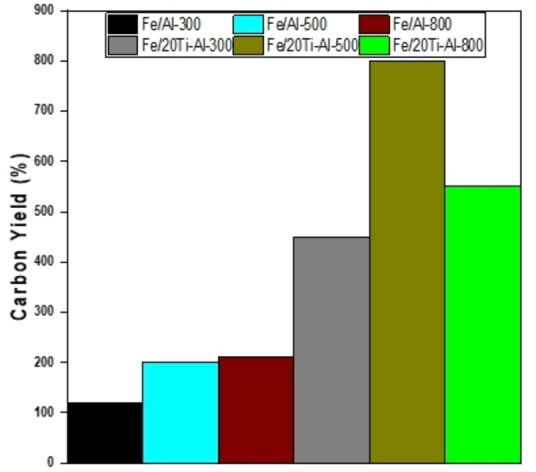
Carbon yield for used iron‐based catalysts loaded on an individual and binary support for reaction time 240 min at 800 °C, CH_4_/N_2_=2 : 1, P=1 atm.

The catalytic activity performance measurements in terms of hydrogen yield and carbon yield are shown in Figure 5 and Figure 6. Figure 5 depicts the experiment results of catalytic methane decomposition at 800 °C and atmospheric pressure.

Fe supported on Al_2_O_3_ and (TiO_2_‐Al_2_O_3_) catalysts with various calcination temperatures for 240 minutes on stream (TOS) is used. The effects of adding TiO_2_ to Al_2_O_3_ and various calcination temperatures were studied. It is apparent from the activity patterns that the introduction of 20 % of TiO_2_ to Al_2_O_3_ has a distinct influence on both the hydrogen and carbon yield. The result showed that the TiO_2_ addition improved the performance of all doped catalysts. For instance, The Fe/(TiO_2_‐Al_2_O_3_) catalysts calcined at 800 °C initially presented hydrogen yields higher than 60 %, which decreased slightly to 52 % due to carbon blockage. A further decrease in calcination temperatures shows a slightly lower initial hydrogen yield of less than 52 %, which starts increasing as the reaction proceeds and reaches about 81 % and 56 % for The Fe/(TiO_2_‐Al_2_O_3_) catalysts calcined at 500 °C and 300 °C respectively after 240 min of CDM reaction as a result of improved the surface area of both catalysts. In contrast, the activity of the Fe/Al_2_O_3_ catalysts calcined at 300, 500, and 800 °C generated a hydrogen yield above 20 % for Fe/Al_2_O_3_‐300, while the hydrogen yield starts from 62 % and 60 % for Fe/Al_2_O_3_‐500 and Fe/Al_2_O_3_‐800 respectively. The catalysts suffered fast deactivation due to the carbon blockage, as seen in Figure S2, and the hydrogen yield decreased to less than 5 % for all the catalysts after 240 minutes of reaction. Table 4 shows some results of a catalyst‘s activity based on iron metal gathered from the literature.

**Table 4:** Comparison of this work activity with previous literature results.

Catalyst	Reaction Temperature^[a]^ (°C)	GV^[a]^	M.C.^[b]^ (%)	HY^[c]^ (%)	Ref.
Fe/MgO	700	15	25	–	[45]
2Ni‐1Fe/1Al	650	42	40	–	[46]
Fe/CeO2	750	1.2	25	–	[47]
Fe/(TiO_2_‐Al_2_O_3_)‐800	800	6	59	52	This work
Fe/(TiO_2_‐Al_2_O_3_)‐500	800	6	87	81	This work
Fe/(TiO_2_‐Al_2_O_3_)‐300	800	6	64	55	This work

[a] GV=GHSV L/g_cat_ h; [b] M.C.=Methane Conversion (%); [c] HY= Hydrogen Yield (%).

Figure 6 shows the carbon yield data for all Fe/(Al_2_O_3_) and Fe/(TiO_2_‐Al_2_O_3_) catalysts calcinated at different temperatures, and it is obvious that carbon yield increases with adding TiO_2_ and reducing calcination temperature.

**Figure 7:**
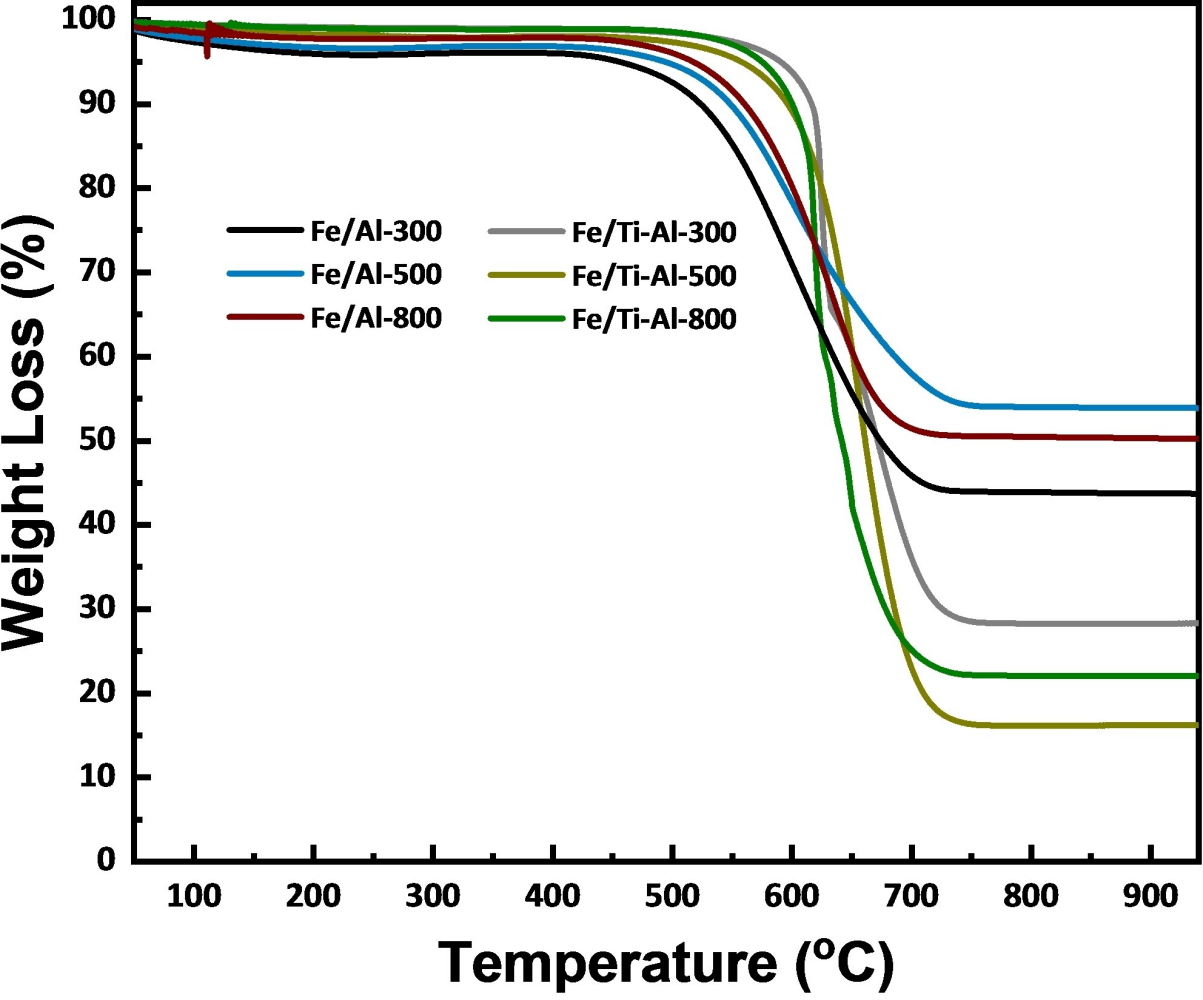
TGA curves for used iron‐based catalysts loaded on an individual and binary support for reaction time 240 min at 800 °C, CH_4_/N_2_=2 : 1, P=1 atm.

For instance, Fe/(TiO_2_‐Al_2_O_3_) catalysts calcined at 800 °C exhibit a carbon yield of 550 % that increases to 800 % when the calcination temperature decreases to 500 °C. At the same time, Fe/(TiO_2_‐Al_2_O_3_) catalysts calcined at 300 °C show a carbon yield of about 450 %; this result matches the TGA analysis trend in Figure 7. On the other hand, Fe/(Al_2_O_3_) catalysts calcined at different temperatures show lower carbon yield. For example, Fe/(Al_2_O_3_) catalysts calcined at 300 °C show a carbon yield of about 120 % and when calcination temperature increased, the carbon yield for Fe/(Al_2_O_3_) catalysts calcined at 500 °C and 800 °C were 200 %, and 210 % respectively, this data are also in line with catalysts activity and TGA analysis as shown in Figure 7. To sum up, the Fe/(TiO_2_‐Al_2_O_3_) catalyst that was calcined at 500 °C was the most effective catalyst for producing hydrogen and carbon. This improved performance can be attributed to a larger surface area that helps to disperse the catalyst and increase the number of active sites.

### Thermo‐Gravimetric Analysis (TGA)

Figure 7 shows the TGA analysis of the spent catalyst operated for four hours. The weight loss percentage indicated the carbon accumulation on the spent catalysts.

Fe/(TiO_2_‐Al_2_O_3_) calcined at 500 °C has the most considerable weight loss of approximately 85 %, while the Fe/(TiO_2_‐Al_2_O_3_) calcined at 800 °C, and Fe/(TiO_2_‐Al_2_O_3_) calcined at 300 °C have the lowest weight loss of roughly 75 % and 70 %, respectively. As previously noted, the Fe/(TiO_2_‐Al_2_O_3_) with various calcination temperatures had the largest relative hydrogen yield. This demonstrates that Fe/(TiO_2_‐Al_2_O_3_) catalysts can accumulate more nanostructure carbon than Fe/Al_2_O_3_ catalysts.

### Raman Spectroscopy

**Figure 8:**
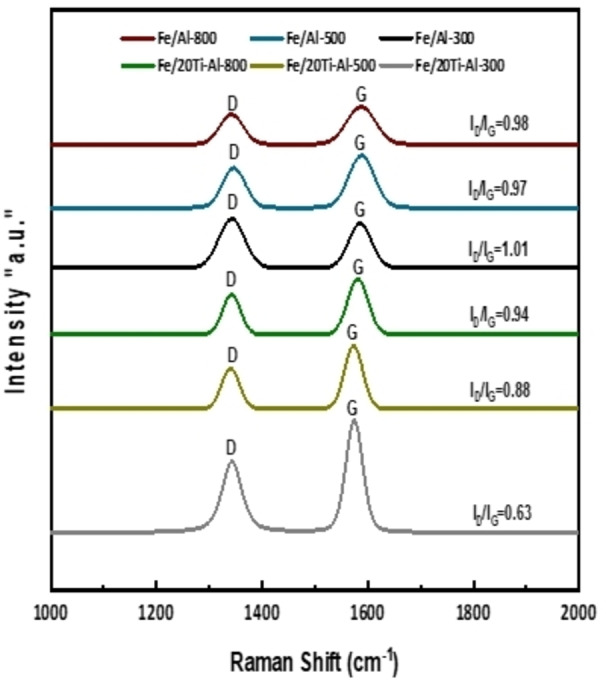
Raman spectra of used iron‐based catalysts loaded on an individual and binary support for reaction time 240 min at 800 °C, CH_4_/N_2_=2 : 1, P=1 atm.

The results of Raman spectroscopy of the catalysts used are shown in Figure 8. All used catalysts showed D and G band peaks. The D band peak at roughly 1350 cm^−1^ was created by disordered carbon, such as amorphous carbon or a damaged graphite sheet. The G‐band peak around 1590 cm^−1^ corresponds to the C−C stretching vibrations characteristic of graphite.^[44]^ The intensity ratio (I_D_/I_G_) of the G and D bands is often used to compare the crystallinity of carbon materials, with a lower (I_D_/I_G_) ratio indicating a higher tendency for the formation of the graphitized carbon.^[44]^

The intensity ratios (I_D_/I_G_) of the Fe/TiO_2_‐Al_2_O_3_‐800, Fe/TiO_2_‐Al_2_O_3_‐500, and Fe/TiO_2_‐Al_2_O_3_‐300 used catalysts were 0.94, 0.88, and 0.63, respectively. In contrast, the intensity ratios (I_D_/I_G_) of the Fe/Al_2_O_3_‐300, Fe/Al_2_O_3_‐500, and Fe/Al_2_O_3_‐800 used catalysts were 1.01, 0.97, and 0.98, respectively, indicating the higher tendency of amorphous carbon formation.

### Transmission Electron Microscopy (TEM)

**Figure 9:**
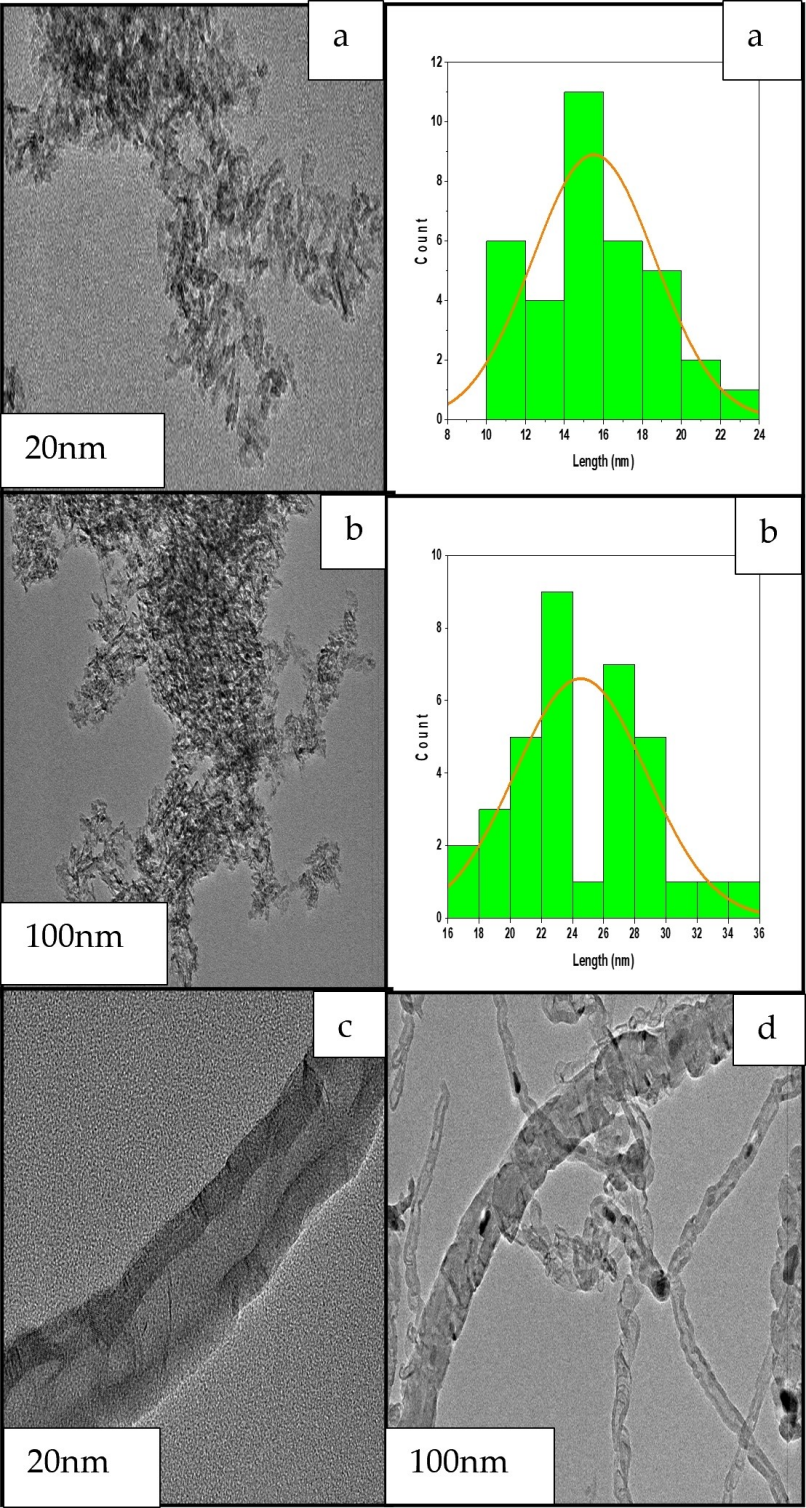
TEM images of the Fe/TiO_2_‐Al_2_O_3_‐500 catalyst before reaction (a, b) and after reaction (c, d).

Figures 9(a‐b) show the TEM images and particle size distributions of the fresh 20Fe/20TiO_2_‐Al_2_O_3_ catalyst at two different magnifications. The catalyst calcined at 500 °C did not show uniform distributions of iron particles. An average particle size distribution of 17.5 nm is obtained. Figures 9(c–d) show TEM images and particle size distributions of the spent 20Fe/20TiO_2_‐Al_2_O_3_ catalyst calcined at 500 °C.

Here, most types of carbon presented are carbon nanotubes, with the active site (iron) being on the tip growth of these nanotubes and no formation of encapsulation carbon. Figure S3 shows that the fresh Fe/Ti‐Al_2_O_3_‐500 exhibits a higher porosity level than the fresh Fe/Al_2_O_3_‐500. This increased porosity significantly enhances mass transfer efficiency and the adsorption of reactants and products throughout the reaction process.

### Temperature‐Programmed Oxidation (TPO)

**Figure 10:**
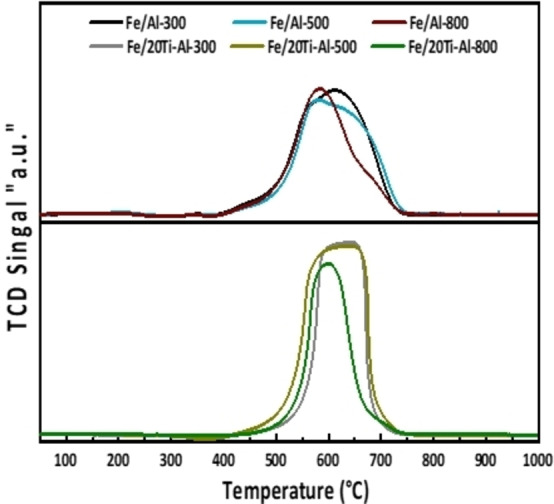
TPO profiles for used iron‐based catalysts loaded on an individual and binary support for reaction time 240 min at 800 °C, CH_4_/N_2_=2 : 1, P=1 atm.

TPO identifies the specific carbon deposits on the surface of the used catalysts. When methane decomposition takes place, it forms different types of carbon on the catalyst‘s surface. These carbon forms can be eliminated by gasification, each at its own distinctive temperature range. For instance, atomic carbon can be gasified at 250 °C, amorphous carbon in the temperature range of 250–600 °C, and graphitic carbon at temperatures exceeding 600 °C. TPO helps us understand and characterize these carbon types based on their gasification temperatures.^[48]^ Figure 10 depicts the TPO profiles of deposited carbon on the respective catalysts.

Most of the peaks indicate the graphitic carbon for the catalysts Fe/(TiO_2_‐Al_2_O_3_) and Fe/Al_2_O_3_, both calcined at (300, 500, and 800 °C). This result agreed with that of the Raman in Figure 8, and it is noted that the most active catalyst had the highest oxidation, which means the amount of carbon on the surface was much more than others, as shown in Figure 7 of TGA.

## Conclusions

The wet‐impregnation approach was used to synthesize Fe‐supported catalysts for catalytic methane decomposition. 20 % titanium oxide‐modified alumina was used as the primary support. The TiO_2_ doping effect of Fe‐containing catalysts was examined. Several characterization approaches were applied to both fresh and spent catalysts. According to the XRD analysis, no diffraction peaks corresponding to the TiO_2_ phase were observed, while in the TPR data, adding titanium oxide to the catalyst composition generated peaks only at 370 to 500 °C. Furthermore, compared to the unmodified alumina‐supported Fe catalyst, the addition of the titanium oxide enhanced the specific surface area by approximately 12 %. The Fe/(TiO_2_‐Al_2_O_3_) calcined at 300 °C, 500 °C, and 800 °C gave the maximum activity and stability, with a hydrogen yield of more than 50 %. Whereas the alumina‐supported Fe catalysts exhibited the lowest yield during the 240 min of TOS. The better performance of the Fe/(TiO_2_‐Al_2_O_3_) catalysts was primarily due to the greater dispersion and higher stabilization of Fe_2_O_3_ and its metallic state in the (TiO_2_‐Al_2_O_3_) support. Alternatively, due to the weak metal‐support interaction, the Fe/Al_2_O_3_ catalysts’ quick deactivation was caused by the agglomeration of Fe_2_O_3_ particles. The TGA results of the Fe/(TiO_2_‐Al_2_O_3_) catalysts demonstrated significant carbon deposits. Furthermore, the TEM results indicated that the carbon deposited on the catalysts’ surface is carbon nanotubes (CNT), and the Raman analysis revealed that the deposited carbon was mainly graphite.

## Experimental Section

### Catalyst Preparation

Iron catalysts were prepared by the incipient impregnation method. Two types of support were tested: alumina and alumina‐titania mixed composite. Loading percent of Fe was fixed at 20 % in this study, where iron precursor Fe(NO_3_)_2_ ⋅ 9H_2_O was dissolved in distilled water under constant stirring at room temperature for 15 min. After that, alumina or alumina titania mixed oxide (80 % alumina‐20 % titania) was gradually added with continuous stirring at 80 °C for 3 hours. The catalysts obtained were dried overnight at 120 °C and finally, Fe/Al_2_O_3_ and Fe/(20TiO_2_‐Al_2_O_3_) calcined at different temperatures, 300 °C, 500 °C, and 800 °C for 5 h. The name of the catalyst is referred to as Fe/Al_2_O_3_‐X and Fe/(TiO_2_‐Al_2_O_3_‐X) for Fe/Al_2_O_3_ and 20Fe/(20TiO_2_‐Al_2_O_3_), respectively, and X state for calcination temperature 300 °C, 500 °C, and 800 °C.

### Catalyst Testing

The catalytic reactions of methane decomposition were carried out under atmospheric pressure at 800 °C in a fixed‐bed tubular reactor (9 mm i.d.) and 300‐mm‐long stainless‐steel tube) inserted in a vertical oven with well‐temperature control, as shown in Figure 1. Initially, 0.15 g of the catalyst was reduced using 30 mL min^−1^ H_2_ at 800 °C for one hour. Thereafter, the system was purged with N_2_ for 15 min to remove any remnant of H_2_. Then, the methane conversion started with the passage of 15 mL min^−1^ of the mixture CH_4_:N_2_ (2 : 1) for 4 hours. Gas chromatography (Shimadzu GC 2004) was utilized to analyze the product gas composition. The GC was connected online and equipped with a thermal conductivity detector (TCD). The type of GC columns used was (Packed Column). The hydrogen yield was calculated using the following equation. 
(2)

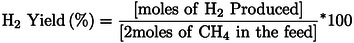



(3)

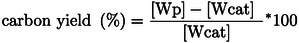




where

Wp is the total weight of the used catalyst.

Wcat is the total weight of the fresh catalyst.

### Catalyst Characterization

The texture properties of the prepared catalysts were determined using a Micro‐meristic Tristar II 3020 surface area and porosity analyzer. For each analysis, 0.2 g of catalyst was degassed at 250 °C for three hours to get rid of moisture content and other adsorbed gases.

The fresh and spent catalysts underwent temperature‐programmed reduction (TPR) and oxidation (TPO) using Micromeritics Auto Chem II 2920. To perform TPR, 70 mg of the catalyst samples were placed in a tube. The sample was pretreated by flushing with argon at 150 °C for an hour and then cooled to room temperature. The furnace temperature was raised to 1000 °C at 10 °C/min in the presence of an H_2_/Ar mixture flowing at 40 mL/min. A cold trap within the machine removed the water produced during the reduction, while a thermal conductivity detector recorded the H_2_ that was being consumed. The TPO measurements were carried out in an oxidative environment to determine the kind of carbon deposited onto the surface of the used catalysts. The spent catalysts were subjected to the same pretreatment as in TPR, and the analysis was done over a temperature range of 50–1000 °C under the flow of 10 % O_2_/He mixture at 40 mL/min.

An X‐ray diffractometer (XRD) was used to examine fresh catalyst samples′ phase formation and crystal structure. A Miniflex Rigaku diffractometer was employed with Cu Kα X‐ray radiation operating at 40 kV and 40 mA to conduct this examination. The diffraction 2θ angle range was set to 10–85° with a step size of 0.01°. The raw data file of the instrument was analyzed using X'pert high score plus software, and the JCPDS data bank was used to match different phases with their corresponding scores. The graphitization degree and the type of carbon deposited over the used catalysts were determined using a JASCO laser Raman spectrometer (NMR‐4500) from (Tokyo, Japan). The spectrometer utilized an excitation beam with a wavelength of 532 nm.

To inspect the morphology of the deposited carbon, TEM measurements of the spent samples were conducted using a JEOL (JEM‐2100F, JEOL Ltd, Tokyo, Japan) transmission electron microscope operated at an accelerating voltage of 120 kV.

The Shimadzu Thermo‐gravimetric analyzer (TGA) was used to determine the quantity of carbon deposits. 10–15 mg of the used catalysts were heated from room temperature to 1000 °C at a heating rate of 20 °C /min, and the machine recorded the weight difference.

## Supporting Information Summary

The supporting information includes XRD patterns of the iron‐based catalysts after refinement by the Rietveld method and SEM images of the Fe/Al_2_O_3_‐500, Fe/Ti‐Al_2_O_3_‐500 catalysts before the reaction, and Fe/Al_2_O_3_‐500 after the reaction.

## Conflict of interests

The authors declare no conflict of interest.

1

## Supporting information

As a service to our authors and readers, this journal provides supporting information supplied by the authors. Such materials are peer reviewed and may be re‐organized for online delivery, but are not copy‐edited or typeset. Technical support issues arising from supporting information (other than missing files) should be addressed to the authors.

Supporting Information

## Data Availability

The data that support the findings of this study are available from the corresponding author upon reasonable request.

## References

[1] L. Zhou , L. R. Enakonda , Y. Saih , S. Loptain , D. Gary , P. Del-Gallo , J. M. Basset , ChemSusChem 2016, 9, 1243–1248. 27159367 10.1002/cssc.201600310

[2] N. Bayat , M. Rezaei , F. Meshkani , Korean J. Chem. Eng. 2016, 33, 490–499.

[3] N. Bayat , M. Rezaei , F. Meshkani , Fuel 2017, 195, 88–96.

[4] Q. Chen , A. C. Lua , Chem. Eng. J. 2020, 389, 124366.

[5] F. Liu , G. Xuan , L. Ai , Q. Liu , L. Yang , Fuel Process. Technol. 2021, 215, 106745.

[6] E. Välimäki , L. Yli-Varo , H. Romar , U. Lassi , C 2021, 7, 50.

[7] L. Alalga , A. Benamar , M. Trari , Int. J. Hydrogen Energy 2021, 46, 28501–28512.

[8] J. Raza , A. H. Khoja , S. R. Naqvi , M. T. Mehran , S. Shakir , R. Liaquat , M. Tahir , G. Ali , J. Environ. Chem. Eng. 2021, 9, 105816.

[9] J. M. Gatica , D. M. Gómez , S. Harti , H. Vidal , Appl. Catal. A 2013, 458, 21–27.

[10] M. Pudukudy , Z. Yaakob , M. S. Takriff , Appl. Surf. Sci. 2015, 356, 1320–1326.

[11] A. I. Osman , N. C. Skillen , P. K. J. Robertson , D. W. Rooney , K. Morgan , Int. J. Hydrogen Energy 2020, 45, 34494–34502.

[12] T. Kreuger , W. P. M. van Swaaij , A. N. R. Bos , S. R. A. Kersten , Chem. Eng. J. 2022, 427, 130412.

[13] S. E. Kim , S. K. Jeong , K. T. Park , K. Y. Lee , H. J. Kim , Catal. Commun. 2021, 148, 106167.

[14] B. A. Al Alwan , M. Shah , M. Danish , M. K. Al Mesfer , M. I. Khan , V. Natarajan , J. Indian Chem. Soc. 2022, 99, 100393.

[15] X. Yang , E. Yang , B. Hu , J. Yan , F. Shangguan , Q. Hao , H. Chen , J. Zhang , X. Ma , J. Environ. Chem. Eng. 2022, 10, 107451.

[16] P. Yan , K. Zhang , Y. Peng , Chem. Eng. Sci. 2022, 250, 117410.

[17] L. Zhou , L. R. Enakonda , M. Harb , Y. Saih , A. Aguilar-Tapia , S. Ould-Chikh , J. Louis Hazemann , J. Li , N. Wei , D. Gary , P. Del-Gallo , J. M. Basset , Appl. Catal. B 2017, 208, 44–59.

[18] S. Kazemi , S. M. Alavi , M. Rezaei , Int. J. Hydrogen Energy 2022, 47, 18370–18383.

[19] S. J. Park , K. D. Kim , Y. S. Park , K. S. Go , W. Kim , M. J. Kim , N. S. Nho , D. H. Lee , J. Ind. Eng. Chem. 2022, 109, 384–396.

[20] J. Wang , L. Jin , Y. Li , H. Hu , Ind. Eng. Chem. Res. 2017, 56, 11021–11027.

[21] V. Ramasubramanian , H. Ramsurn , G. L. Price , Int. J. Hydrogen Energy 2020, 45, 12026–12036.

[22] H. Sun , S. Ren , X. Ji , W. Song , Q. Guo , B. Shen , Int. J. Hydrogen Energy 2023, 48, 13081–13096.

[23] J. Alves Silva , J. B. Oliveira Santos , D. Torres , J. L. Pinilla , I. Suelves , Int. J. Hydrogen Energy 2021, 46, 35137–35148.

[24] M. Karaismailoğlu , H. E. Figen , S. Z. Baykara , Int. J. Hydrogen Energy 2020, 45, 34773–34782.

[25] A. H. Fakeeha , S. O. Kasim , A. A. Ibrahim , A. S. Al-Awadi , E. Alzahrani , A. E. Abasaeed , A. E. Awadallah , A. S. Al-Fatesh , Front. Chem. 2020, 8, 527057. 10.3389/fchem.2020.00317 PMC7201101 32411666

[26] U. P. M. Ashik , W. M. A. Wan Daud , H. F. Abbas , Renewable Sustainable Energy Rev. 2015, 44, 221–256.

[27] Y. Shen , A. C. Lua , Int. J. Hydrogen Energy 2015, 40, 311–321.

[28] G. Yergaziyeva , N. Makayeva , A. Abdisattar , M. Yeleuov , S. Soloviev , M. Anissova , A. Taurbekov , K. Dossumov , E. Akkazin , C. Daulbayev , Chem. Pap. 2022, 76, 7405.

[29] J. L. Pinilla , R. Utrilla , R. K. Karn , I. Suelves , M. J. Lázaro , R. Moliner , A. B. García , J. N. Rouzaud , Int. J. Hydrogen Energy 2011, 36, 7832–7843.

[30] B. Zapata , M. A. Valenzuela , J. Palacios , E. Torres-Garcia , Int. J. Hydrogen Energy 2010, 35, 12091–12097.

[31] R. Guil-Lopez , J. A. Botas , J. L. G. Fierro , D. P. Serrano , Appl. Catal. A 2011, 396, 40–51.

[32] J. Zhang , L. Jin , H. Hu , Y. Xun , Fuel 2012, 96, 462–468.

[33] A. I. Alharthi , E. Abdel-Fattah , M. A. Alotaibi , M. N. Al-Shalwi , Int. J. Energy Res. 2022, 46, 17497–17510.

[34] L. Zhou , L. R. Enakonda , Y. Saih , S. Loptain , D. Gary , P. Del-Gallo , J. M. Basset , ChemSusChem 2016, 9, 1243–1248. 27159367 10.1002/cssc.201600310

[35] A. E. Awadallah , A. A. Aboul-Enein , D. S. El-Desouki , A. K. Aboul-Gheit , Appl. Surf. Sci. 2014, 296, 100–107.

[36] T. Wu , X. Zhu , Z. Xing , S. Mou , C. Li , Y. Qiao , Q. Liu , Y. Luo , X. Shi , Y. Zhang , X. Sun , Angew. Chem. Int. Ed. 2019, 58, 18449–18453. 10.1002/anie.201911153 31549471

[37] G. Shen , R. Zhang , L. Pan , F. Hou , Y. Zhao , Z. Shen , W. Mi , C. Shi , Q. Wang , X. Zhang , J. J. Zou , Angew. Chem. Int. Ed. 2020, 59, 2313–2317. 10.1002/anie.201913080 31743560

[38] H. M. Bian , Y. Yang , Y. Wang , W. Tian , H. F. Jiang , Z. J. Hu , W. M. Yu , J. Alloys Compd. 2012, 525, 63–67.

[39] A. Guidara , K. Chaari , J. Bouaziz , J. Mater. Sci. Technol. 2012, 28, 1130–1136.

[40] O. Phichairatanaphong , Y. Poo-Arporn , M. Chareonpanich , W. Donphai , ACS Omega 2022, 7, 14264–14275. 35573207 10.1021/acsomega.2c01016 PMC9089693

[41] A. S. Al-Fatesh , A. H. Fakeeha , A. A. Ibrahim , W. U. Khan , H. Atia , R. Eckelt , K. Seshan , B. Chowdhury , J. Saudi Chem. Soc. 2018, 22, 239–247.

[42] Y.-H. Wang , H.-M. Liu , B.-Q. Xu , J Mol Catal A Chem 2009, 299, 44–52.

[43] M. A. Ermakova , D. Y. Ermakov , Catal. Today 2002, 77, 225–235.

[44] B. Gao , I. W. Wang , L. Ren , J. Hu , Energy Fuels 2019, 33, 9099–9106.

[45] A. E. Awadallah , M. S. Abdel-Mottaleb , A. A. Aboul-Enein , M. M. Yonis , A. K. Aboul-Gheit , Chem. Eng. Commun. 2015, 202, 163–174.

[46] N. Bayat , M. Rezaei , F. Meshkani , Int. J. Hydrogen Energy 2016, 41, 1574–1584.

[47] L. Tang , D. Yamaguchi , N. Burke , D. Trimm , K. Chiang , Catal. Commun. 2010, 11, 1215–1219.

[48] A. A. Ibrahim , A. H. Fakeeha , A. S. Al-Fatesh , A. E. Abasaeed , W. U. Khan , Int. J. Hydrogen Energy 2015, 40, 7593–7600.

