# Supplementary material for: Hydrogen Production via Methane Decomposition over Alumina Doped with Titanium Oxide‐Supported Iron Catalyst for Various Calcination Temperatures
Source: ChemistryOpen. 2023 Dec 12;13(4):e202300173. doi: 10.1002/open.202300173 (PMC11004458; doi:10.1002/open.202300173)
Supplement: Supplementary file 1 — Supporting Information [file OPEN-13-e202300173-s001.pdf]

ChemistryOpen

Supporting Information

Hydrogen Production via Methane Decomposition over Alumina Doped with Titanium Oxide-Supported Iron Catalyst for Various Calcination Temperatures

Hamid Ahmed, Mohammed F. Alotibi,* Anis H. Fakeeha, Ahmed A. Ibrahim, Ahmed E. Abasaeed, Ahmed I. Osman,* Abdulrahman S. Al-Awadi, Naif Alarifi, and Ahmed S. Al-Fatesh*

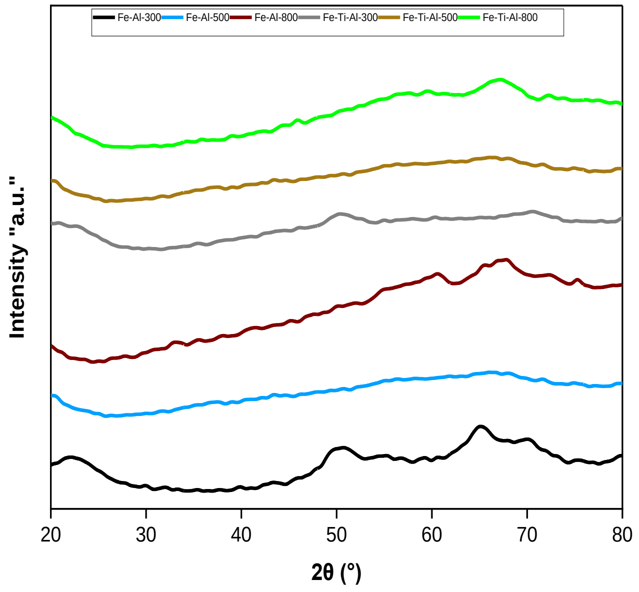

Figure S1-a XRD patterns of the iron-based catalysts after refinement by the Rietveld method

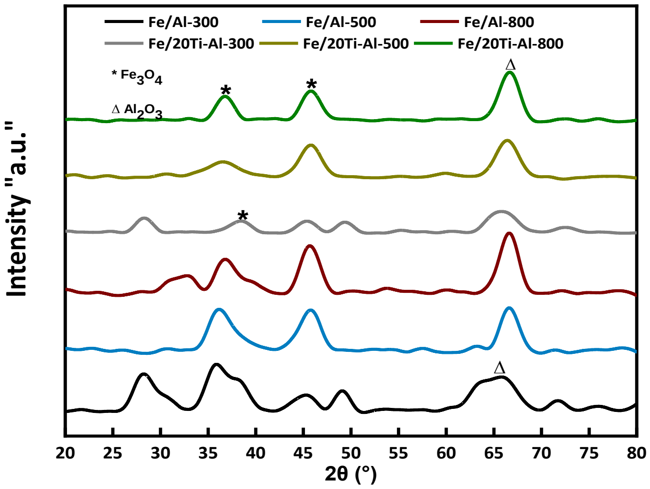

Figure S1-b XRD patterns of the iron-based catalysts before refinement by the Rietveld method

Upon careful observation of Figure S1, it is evident that there are no discernible peaks associated with TiO_2 following the refinement process using the Rietveld method. This observation strongly suggests that the TiO_2 particles are effectively dispersed.

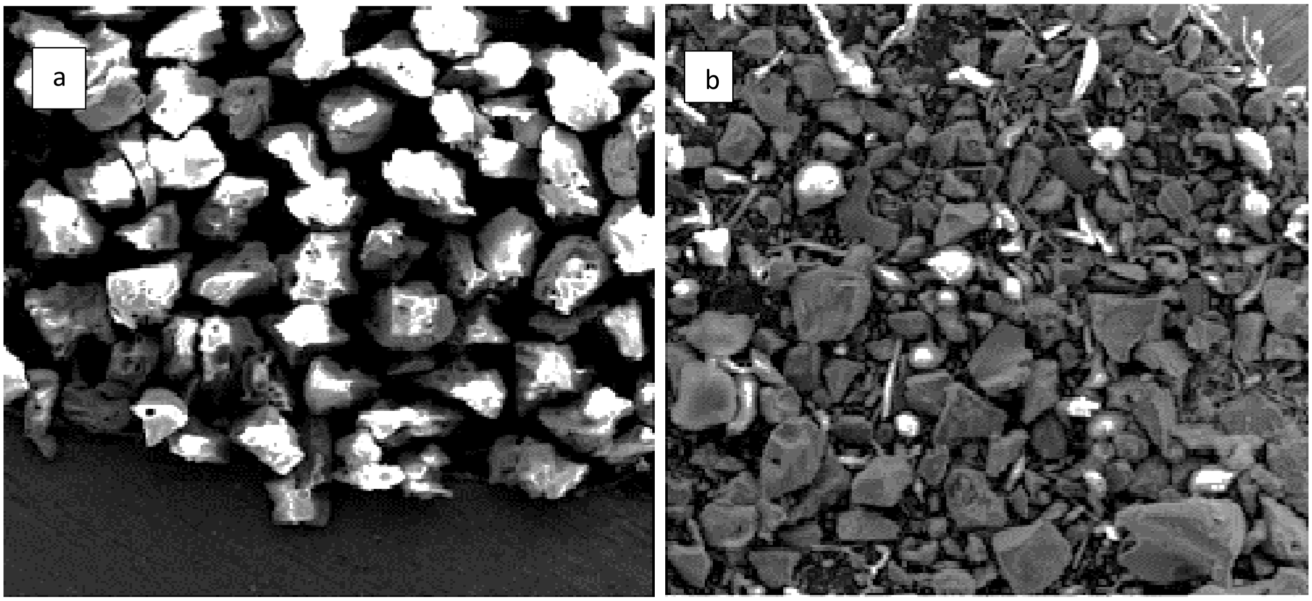

Figure S2. SEM images of the Fe/Al_2O_3-500 catalyst before reaction (a) and after reaction (b).

The SEM images (Figure S2) of both fresh and spent Fe/Al_2O_3-500 catalysts were captured to gain insights into the morphology and structural changes that occurred during the reaction. These images can be used to confirm the presence of carbon and its impact on catalyst deactivation.

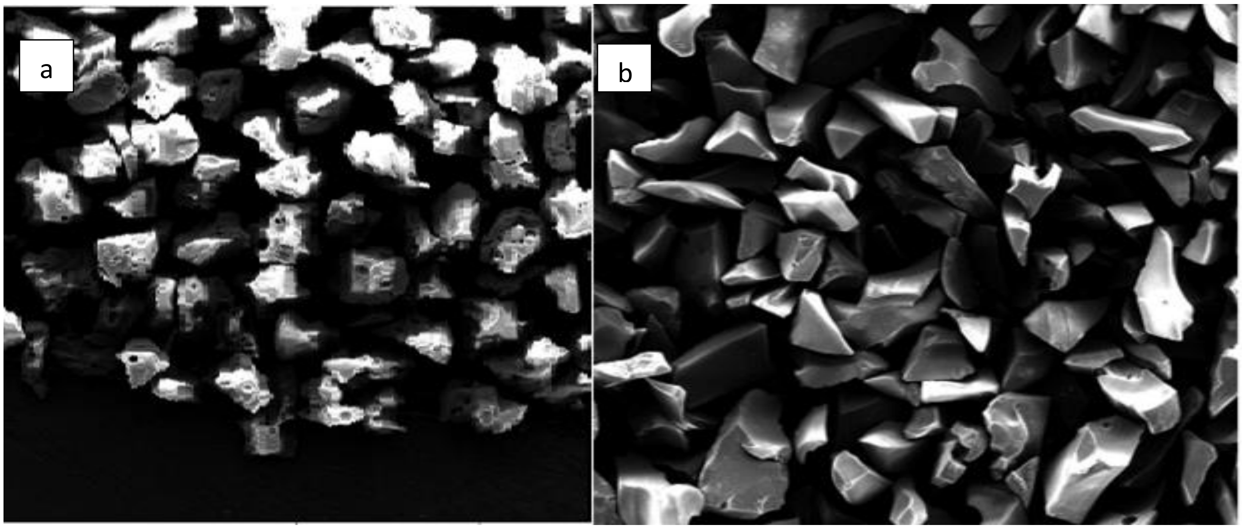

Figure S3. SEM images of the fresh Fe/Al_2O_3-500 catalyst (a) and the fresh $Fe/Ti-Al_2O_3$-500 catalyst (b)

Figure S3 shows that the fresh $Fe/Ti-Al_2O_3$-500 exhibits a higher porosity than the fresh Fe/Al_2O_3-500. This increased porosity significantly enhances mass transfer efficiency and the adsorption of reactants and products throughout the reaction process.
